# Natural Eutectic Solvent-Based Temperature-Controlled Liquid–Liquid Microextraction and Nano-Liquid Chromatography for the Analysis of Herbal Aqueous Samples

**DOI:** 10.3390/foods14010028

**Published:** 2024-12-25

**Authors:** Álvaro Santana-Mayor, Giovanni D’Orazio, Miguel Ángel Rodríguez-Delgado, Bárbara Socas-Rodríguez

**Affiliations:** 1 Departamento de Química, Área de Química Analítica, Facultad de Ciencias, Universidad de La Laguna (ULL), Avenida Astrofísico Francisco Sánchez s/n, 38206 San Cristóbal de La Laguna, Tenerife, Spain; mrguez@ull.edu.es; 2 Istituto per i Sistemi Biologici (ISB), CNR-Consiglio Nazionale delle Ricerche, Montelibretti, 00015 Rome, Italy; giovanni.dorazio@cnr.it

**Keywords:** green solvent, deep eutectic solvent, liquid–liquid microextraction, nano-liquid chromatography, green chemistry, miniaturization, food, beverage

## Abstract

In this work, two novel (-)-menthol-based hydrophobic natural eutectic solvents with vanillin and cinnamic acid were prepared and applied as extraction solvents. In this regard, 12 endocrine disruptors, including phenol, 2,4-dimethylphenol, 2,3,6-trimethylphenol, 4-*tert*-butylphenol, 4-*sec*-butylphenol, 4-*tert*-amylphenol, 4-*n*-hexylphenol, 4-*tert*-octylphenol, 4-*n*-heptylphenol, 4-*n*-octylphenol, and 4-*n*-nonylphenol and bisphenol A, were studied in a green tea drink. A temperature-controlled liquid–liquid microextraction was used as the extraction method, and nano-liquid chromatography–ultraviolet detection was used as the separation and determination system. Different parameters affecting the compatibility of the non-ionic eutectic solvents with water-polar organic solvent mixtures and chromatographic and detection systems were optimized, including injection/dilution solvent, injection mode, mobile phase composition, and step gradient. With the same purpose, two stationary phases were tested, including XBridge^®^ C_18_ and a mixed-phase Cogent C_30_-XBridge^®^ C_18_. Finally, the greenness and blueness of the methodology were assessed to evaluate the environmental profile and usability of the procedure.

## 1. Introduction

Sustainability entails satisfying current requirements without compromising the ability of future generations to meet their own [1]. In practical terms, it involves a balance between economic growth, environmental protection, and social well-being. Chemistry can play a crucial role in this topic due to its involvement in various key aspects such as chemical synthesis, resource management, waste minimization, and improvement of industrial processes. It focuses on developing methods and chemicals that reduce or eliminate the use of hazardous substances, minimize energy and resource consumption, and decrease waste generation [2,3,4]. In this regard, the field of analytical chemistry can provide tools and techniques for monitoring and assessing environmental quality, controlling pollution, ensuring the safety of food and chemicals, and optimizing processes to minimize waste and maximize efficiency. Furthermore, analytical chemistry is employed in the development of recycling processes and in the characterization of materials to improve resource efficiency [4].

The concept of “*white analytical chemistry*” is introduced as an approach to harmonize the principles of green analytical chemistry with functionality [2,5]. This idea aligns well with the broader principles of sustainability, which involve balancing environmental impact with practical effectiveness. The principles of sustainability emphasize the minimization of waste, energy consumption, and the use of hazardous substances while also promoting efficiency and effectiveness. Similarly, white analytical chemistry aims to achieve analytical goals while minimizing environmental impact. Nevertheless, the sustainability of an analytical method relies on a multitude of parameters, including analytical efficiency, safety, and eco-friendliness, alongside practicality and economic considerations, without favoring one over the others [2]. This entails achieving a thoroughly balanced method. However, this task is not a simple one due to the numerous challenges involved in transitioning from traditional chemistry to a cleaner and more sustainable alternative while ensuring analytical results and the usefulness and functionality of the method.

Hence, implementing different strategies aimed at advancing analytical chemistry toward greater sustainability and integrity while ensuring continued efficiency and functionality is currently a hot topic. Some of the main approaches involve reducing the volume of solvents and reagents used in analytical procedures, optimizing energy consumption in instrumentation, and selecting safer alternatives to hazardous chemicals. These practices align with the goals of sustainability by conserving resources, reducing pollution, and enhancing safety.

In this regard, natural deep eutectic solvents (NADESs) represent a promising solution in the development of green and environmentally friendly methodologies for chemical analysis [6]. NADESs offer several features that align with the principles of sustainability. These solvents are composed of cheap and readily available natural components. They are considered greener alternatives to conventional organic solvents due to their low toxicity, biodegradability, and low volatility. These materials provide great versatility since they can be tailored to specific applications by choosing appropriate components, allowing for a wide range of solvent properties such as polarity, viscosity, and conductivity. This versatility enables their use in various analytical techniques, including extraction and chromatography, among others [7,8]. NADESs have shown promising features in improving analytical performance compared to traditional solvents. They can provide better solubility for certain analytes, enhance selectivity, and improve extraction efficiencies, leading to more sensitive and accurate analytical methods [7,8,9,10]. A subclass of NADESs is constituted by those solvents composed of naturally occurring hydrophobic compounds, such as terpenes and long-chain fatty acids, known as hydrophobic NADESs (HNADESs) [11,12]. These solvents exhibit hydrophobic characteristics, making them particularly useful for applications in which water immiscibility is desired. In addition, they offer a more sustainable alternative to conventional organic solvents, reducing environmental impact and health risks when compared, for instance, with traditional chlorinated solvents used in liquid–liquid microextraction (LLME) procedures. While being compliant with green chemistry principles (renewable, biodegradable, and non-toxic components), this kind of NADES can selectively dissolve hydrophobic compounds, enabling efficient extraction or separation of target analytes from aqueous solutions. This high selectivity can enhance the sensitivity and accuracy of analytical methods. Compared to volatile organic solvents, HNADESs typically exhibit lower volatility, minimizing solvent loss and reducing the risk of exposure to harmful vapors in laboratory settings. However, despite the many advantages of NADESs, particularly of HNADESs, these solvents often exhibit higher viscosity compared to conventional solvents, which can hinder mass transfer and affect the efficiency of extraction or separation processes [11]. This aspect can also be a challenge when integrated with analysis systems. High viscosity may also lead to increased backpressure in chromatographic systems, affecting system performance and column longevity [13]. Therefore, the optimization of operating conditions may be required to mitigate viscosity-related challenges and other limitations, including solvent compatibility. Addressing these limitations requires ongoing research and development efforts to optimize the performance and applicability of HNADESs in analytical systems and applications. Despite all the features mentioned above, and as stated in some critical articles about eutectic solvents (ESs) [7,14,15,16,17], the definition of these solvents cannot be given only by their liquid state at the working temperature. The need to fully characterize eutectic mixtures in order to check whether they are (NA)DESs or (NA)ESs is becoming more and more evident.

Alkylphenol ethoxylates are among the most extensively utilized non-ionic surfactants. Their amphiphilic structure enables these compounds to form micelles in aqueous solutions, facilitating the removal of fats from surfaces into water and making them ideal for cleaning products. Additionally, they find applications in diverse industries such as paper and pulp production, textiles, latex paints, phytosanitary products, and plastic manufacturing, among others. The most common ones are nonylphenol and octylphenol ethoxylates [18,19,20]. In the environment, alkylphenol ethoxylates undergo degradation to yield shorter-chain ethoxylates and other derivatives, including alkylphenols, among other by-products. Apart from serving as raw materials for ethoxylate production, alkylphenols are utilized in the manufacture of resins and polymers as thermal stabilizers, as well as antioxidants [18,19]. However, these metabolites exhibit higher toxicity, persistence, and bioaccumulation compared to their precursors. Furthermore, they can mimic the action of natural hormones, becoming potential endocrine disruptors and leading to adverse effects on reproduction and health. Given their widespread presence and persistence, there is concern about potential overexposure to these xenobiotic compounds, primarily through the consumption of contaminated food or water [18,19,20]. Numerous studies have reported their presence in several matrices, including environmental water, sediments, soil, food, drinking water, and even human tissues [18].

Bisphenol A (BPA) stands out as the most significant synthetic compound globally, given its substantial annual production and widespread utilization [21,22]. BPA is extensively utilized in the manufacturing of polymers and resins, which serve as essential components in the production of various plastic materials and other chemical compounds. Consequently, BPA is ubiquitous in consumer goods such as food packaging, containers, thermal paper, inks, textiles, electronic devices, and various industrial applications [21,22,23]. In addition, BPA is recognized for its capacity to disrupt endocrine function, particularly impacting the action of endogenous estrogenic steroid hormones [23] and other hormones, including leptin, insulin, and thyroxin [22,24]. Consequently, BPA exposure can lead to several health issues, such as hormonal imbalances, reproductive disorders, and developmental problems, as well as mutagenic and carcinogenic effects [25,26,27,28,29,30,31]. The primary route of exposure to BPA is through diet [32,33], but it also can leach into food products, with factors such as pH, fat content, temperature, and contact time influencing the extent of this process [34].

To mitigate the risks associated with alkylphenols and bisphenols, there have been regulatory measures to restrict or ban their use in certain applications. Therefore, the development of analytical methodologies to determine these compounds in food matrices is necessary to ensure food safety and consumer health.

Several analytical procedures have been developed for the extraction and determination of alkylphenols and BPA, including traditional extraction techniques such as liquid–liquid extraction, solid–liquid extraction, or solid-phase extraction, and miniaturized approaches including (magnetic) micro-solid phase extraction, solid-phase microextraction, stir bar sorptive extraction, LLME, hollow-fiber, and single drop microextraction, among others [21,35,36,37]. Regarding the separations and determination systems, most of the analyses were developed in liquid chromatography (LC)-based systems coupled to conventional detectors such as UV, diode-array, or fluorescence detectors and (tandem) mass spectrometry systems, although few based on gas-chromatography can be found [18,36,37]. Regarding the use of NAES for alkylphenols and BPA extraction, some applications are found in the literature for the analysis of environmental waters [38,39,40,41], polyethylene-packed injection solutions [42], and food [43,44,45,46,47] by (ultra-)high-performance LC [38,39,40,44,45,46,47] hyphenated with diode-array [39,40,41,43], UV [38], fluorescence detection [47], or tandem mass spectrometry systems [44,45,46] and gas chromatography–mass spectrometry [42]. In addition, only two articles analyze these compounds using miniaturized analysis techniques [48,49]. However, despite the fact that the combination of deep eutectic solvents with these analysis systems has been previously reported [50,51], none of them combined a green extraction agent based on hydrophobic NAES (HNAES) with nano-LC-UV detection for the analysis of alkylphenols and BPA.

Therefore, this work aims to develop a sustainable analytical methodology based on HNAES-LLME combined with nano-LC-UV detection for the analysis of alkylphenols (phenol, 2,4-dimethylphenol (2,4-DMP), 2,3,6-trimethylphenol (2,3,6-TMP), 4-*tert*-butylphenol (4-TBP), 4-*sec*-butylphenol (4-SBP), 4-*tert*-amylphenol (4-TAP), 4-*n*-hexylphenol (4-HexP), 4-*tert*-octylphenol (4-TOP), 4-*n*-heptylphenol (4-HepP), 4-*n*-octylphenol (4-OP), and 4-*n*-nonylphenol (4-NP)) and BPA in tea aqueous samples. The prepared HNAES based on the terpene (-)-menthol and vanillin or *trans*-cinnamic acid have not been previously reported. These HNAES have been completely characterized using Fourier Transform Infrared (FT-IR) spectroscopy and differential scanning calorimetry (DSC) to perform the solid–liquid phase diagrams. In addition, to the best of our knowledge, this work constitutes the first approach that combines HNAES with nano-LC-based separation. Finally, different analytical metric tools were used to evaluate the greenness and functionality of the methodology.

## 2. Materials and Methods

### 2.1. Chemicals and Materials

All standards and solvents were used as received without further purification. Analytical standards, including phenol (CAS 108-95-2), BPA (CAS 80-05-7), 2,4-DMP (CAS 105-67-9), 2,3,6-TMP (CAS 2416-94-6), 4-TAP (CAS 80-46-6), 4-OP (CAS 1806-26-4), 4-TOP (CAS 140-66-9), 4-NP (CAS 104-40-5), with purities exceeding 96.3% (*w*/*w*), were sourced from Dr. Ehrenstorfer GmbH (Augsburg, Germany). 4-TBP (CAS 98-54-4), 4-HexP (CAS 2446-69-7), and 4-HepP (CAS 1987-50-4) with a purity higher than 99.9% (*w*/*w*) with purities above 99.9% (*w*/*w*), were obtained from Sigma-Aldrich Chemie (Madrid, Spain). 4-SBP (CAS 99-71-8), with a purity of 98.0% (*w*/*w*), was acquired from Combi-Blocks, Inc. (San Diego, CA, USA). Di-*n*-hexyl phthalate-3,4,5,6-d_4_ (DHP-d_4_, 97% (*w*/*w*) purity, CAS 1015854–55-3, Dr. Ehrenstorfer GmbH, (Augsburg, Germany) served as the surrogate internal standard. Benzene, ethylbenzene, and propylbenzene, used as test mix for chromatographic performance, were supplied by Merck (Darmstadt, Germany). Alkylphenols and BPA stock solutions were prepared in methanol at 1000 mg/L and stored at –18 °C in darkness. Working solutions were prepared by dilution on the corresponding solvent.

HPLC-grade water, methanol, acetonitrile (ACN), and acetone (HiPerSolv CHROMANORM^®^) were obtained from VWR International (Milan, Italy). Vanillin was purchased from Sigma-Aldrich (Milan, Italy), while (-)-menthol and *trans*-cinnamic acid were sourced from Fluka (Milan, Italy).

Polyimide-coated fused silica capillaries (375 µm outer diameter, 100 µm inner diameter) were procured from Polymicro Technologies (Phoenix, AZ, USA) to fabricate capillary columns. Two stationary phases were evaluated: XBridge^®^ C_18_ (3.5 µm, 130 Å, 18% carbon load) obtained from a Waters (Milford, MA, USA) guard cartridge and Cogent C_30_™ (3 µm, 200 Å, 18% carbon load) purchased in bulk from MicroSolv (Leland, NC, USA).

### 2.2. Sample Selection

A commercially available non-alcoholic green tea beverage was purchased from local supermarkets in Rome, Italy. The beverage primarily consists of green tea infusion (water and green tea), sugar, and concentrated peach and lemon juices.

### 2.3. Apparatus and Software

A nano/capillary LC-pump, Ultimate™ Capillary HPLC unit (LC Packings Dionex, Amsterdam, The Netherlands), equipped with a manual injector and an internal flow splitter, was used for all experiments. A low-dispersion six-port valve (VICI VALCO Instrumentations, Houston, TX, USA) with a 15 µL external loop served as the injection device. To minimize band broadening, capillary columns were directly connected to the injector valve.

A step gradient mode, controlled by the injection valve, was employed for analyte separation. The external loop was used for both sample loading and mobile phase introduction. Injection volumes were adjusted based on the column workflow. In this setup, ACN was exclusively used as the pump solvent to propel the injection plug into the column. The waste solvent was recycled into the pump reservoir [52]. To achieve step gradient conditions, plug injections of aqueous/organic mixtures with increasing elution strength were performed. The specific gradient profile is outlined in Table 1. The injection sequence consisted of first conditioning the capillary column with an ACN/water (50/50 (*v*/*v*)) mixture. Then, a water plug was injected for 10 s. Subsequently, the sample was injected for another 10 s (40 nL), followed by the step gradient. Finally, the initial conditions were re-established for a total run time of 18 min.

On-column detection at 214 nm was employed for analysis. The chromatographic system was maintained at room temperature (18–22 °C). A Chromeleon™ Chromatography Management System (Version 6.6, LC Packings) controlled the pump and UV detector.

During column packing, an FS 100b Decon ultrasonic bath (King of Prussia, PA, USA) was used to sonicate mobile phases, analytes, and stationary phase slurries. An optical transmission microscope (Stereozoom 4, Bausch & Lomb, Bridgewater, NJ, USA) was used to monitor the packing process. A PerkinElmer Series 10 HPLC pump (Waltham, MA, USA) was utilized for packing fused silica capillary columns.

FT-IR spectra of pure compounds and HNAESs were acquired using an Agilent Cary 630 instrument (Santa Clara, CA, USA) equipped with a diamond attenuated total reflectance module and KBr beam splitter. Spectra were recorded from 4500 to 400 cm^−1^ at 4 cm^−1^ resolution, with 256 scans. DSC measurements were performed on a TA Instruments Discovery DSC 25 (New Castle, DE, USA) with a Refrigerated Cooling System 90. Samples were analyzed under nitrogen at a 50 mL/min flow rate in hermetic Tzero aluminum pans. Heating cycles ranged from −80 °C to 150 °C at a rate of 10 °C/min. Additional characterization details and discussion can be found in the Supplementary Material (Characterization of HNAES Section and Figures S1–S3).

### 2.4. Preparation of Capillary Columns

All capillary columns were prepared in the laboratory using a slurry packing method [53], as previously reported. Capillaries were packed to an effective length of 25.0 cm and a total one of 35.0 cm. For the scheme of the system used for capillary packaging, see ref. [51].

Approximately 35–40 cm of fused silica capillary was connected to a mechanical frit and a stainless steel HPLC pre-column reservoir. A 50 mg stationary phase slurry in 1 mL of acetone was sonicated and pumped through the capillary at 35 MPa (350 bar) to achieve a 30 cm packed bed. The packing process was ultrasound-assisted, ensuring a uniform stationary phase structure. After washing with water and conditioning with 5 mM NaCl, inlet and outlet frits were created by electrically heating a small section at 700 °C for 7–8 s. The temporary frit was removed, and the excess stationary phase was purged [51]. A mixed-phase column was prepared by packing 1.5 cm of Cogent C_30_ and 23.5 cm of XBridge^®^ C_18_. Detection windows were created by removing the polyimide layer near the outlet frit, resulting in a 26.6 cm effective length. Columns were equilibrated with a 50/50 ACN/water mixture.

A test mixture of benzene, ethylbenzene, and propylbenzene (0.1% *v*/*v* in water) was injected at 60 nL and separated isocratically using an 80/20 ACN/water mobile phase to evaluate the chromatographic performance of each column. Key chromatographic parameters were compared to assess system robustness and column quality.

### 2.5. HNAES Preparation

The HNAESs evaluated were prepared in glass vials by magnetic stirring of the components in a glycerin bath at 60 °C and 800 rpm for 30 min at specific molar ratios. For this purpose, the AREX Heating Magnetic Stirrer with a digital thermoregulator from VELP Scientifica Srl (Monza and Brianza, Italy) was used. The obtained eutectic mixtures were stored in a vacuum desiccator until use to avoid moisture absorption. The components used for HNAES preparation and their thermal properties are listed in Table 2.

### 2.6. Temperature-Controlled Liquid–Liquid Microextraction Procedure

**Figure 1:**
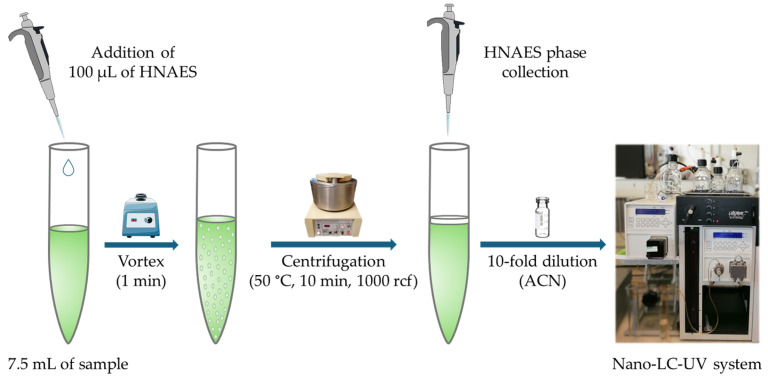
Schematic illustration of the temperature-controlled LLME-nano-LC-UV procedure.

A 7.5 mL aliquot of spiked or non-spiked tea sample at 50 °C was transferred into a 10 mL glass centrifuge tube. Then, 100 µL of HNAES at 50 °C (heated in a Techne^®^ Dri-Block^®^ heater DB-2A, Minneapolis, MN, USA) was added to the sample and vortexed for 1 min. At this stage, a cloudy solution of micro-droplets of HNAES was formed in the aqueous sample. Afterward, it was centrifuged at 1000 rcf for 10 min at 50 °C to achieve phase separation. Then, 10 µL of the HNAES upper enriched phase containing target analytes was collected and transferred into an injection vial containing 90 µL of ACN (10-fold dilution). Next, 40 nL were injected into the nano-LC-UV system in triplicate (see Figure 1).

### 2.7. Sustainability Assessment

Several tools have been proposed to assess the compliance of metric tools with the principles of green analytical chemistry. These include the National Environmental Methods Index (NEMI) [57], the Analytical Eco-Scale [58], the (Complementary) Green Analytical Procedure Index (GAPI) [59,60], and the Analytical GREEnness metric approach (AGREE) [61,62]. Each of these methods offers unique considerations and frameworks for evaluating their eco-friendliness. Despite the criticism that these greenness assessment tools lack a comprehensive assessment of methods, thus falling short in evaluating them against sustainability [2], they provide a simple view of the green profile of the technique. In addition, with the recent introduction of the Blue Applicability Grade Index (BAGI) [63] approach as a measure of the practicability of the methodology, not only the greenness of the method is outlined, but also its practical aspects. Therefore, it represents a simple way to identify the weaknesses and strengths of the method but highlights that even though it can be better or worse in its green and blue profile, it must continue to perform in analytical results. In this work, to facilitate comparative analysis of these tools and the interpretation of their results, a comparison specifically focusing on the AGREE [61] and BAGI [63] metric tools was conducted.

## 3. Results and Discussion

### 3.1. Optimization of Nano-LC UV Method

As previously reported [51], alkylphenols are characterized by their non-charged state over a wide pH range, with pKa values exceeding 9.5, and possess a hydrophobic molecular structure typified by partition coefficients (log P) ranging from 1.46 to 5.76 [64]. These properties suggest limitations on the selection of the stationary phase, especially concerning the solvent used to dissolve the mixture of these compounds. In light of an environmental perspective, a reverse-phase partitioning mechanism was established, opting for stationary phases tailored to enhance interactions with the target compounds. Accordingly, based on conditions previously optimized [51], two reverse-phase stationary phases—one comprising C_18_ and one with C_30_-C_18_ combination bound to silica particles, all completely end-capped—were investigated. Various parameters (such as mobile phase composition and injection solvent) were systematically modified to optimize chromatographic separation in terms of both performance and analysis duration.

In general terms, nano-LC offers many advantages as a separation technique. In addition to the very low flow rates (nanoliters per minute order), nano-LC provides low chromatographic dilution [65] and better packing bed homogeneity, improving the expected chromatographic band broadening [66]. The low injection volume (nanoliters) that limit the sensitivity of the method can be improved by increasing the injection time by working in overloading mode. Therefore, the key challenge in this aspect is based on the control of chromatographic conditions for an on-column focusing approach, in which the analytes are on-column pre-concentrated [67].

In reverse-phase chromatography, injecting non-polar compounds into weak mobile phases can lead to band broadening at the column head. Adding water to the solvent dilution offers a simple method to concentrate and focus analytes, mitigating band broadening and improving chromatographic resolution [68]. On the contrary, injecting organic solvents (such as ACN) has led to inferior chromatographic profiles, despite using small volumes (e.g., 40 nL). Notably, the most significant compression of the injection plug was achieved with water as the dilution solvent [68]. Given the low solubility of alkylphenols in addition to the hydrophobic profile of the extraction solvent, 100% organic solvent was used during optimization. In this regard, the chromatographic separation was focused on the combination of reverse-phase chromatography with an injection solvent with the maximum elution strength. While increasing solubility aids compound dissolution, it can negatively impact chromatographic separation. A balance must be struck to ensure both solubility and chromatographic performance. Failure to do so can result in decreased efficiency, resolution, and impaired on-column focusing.

**Figure 2:**
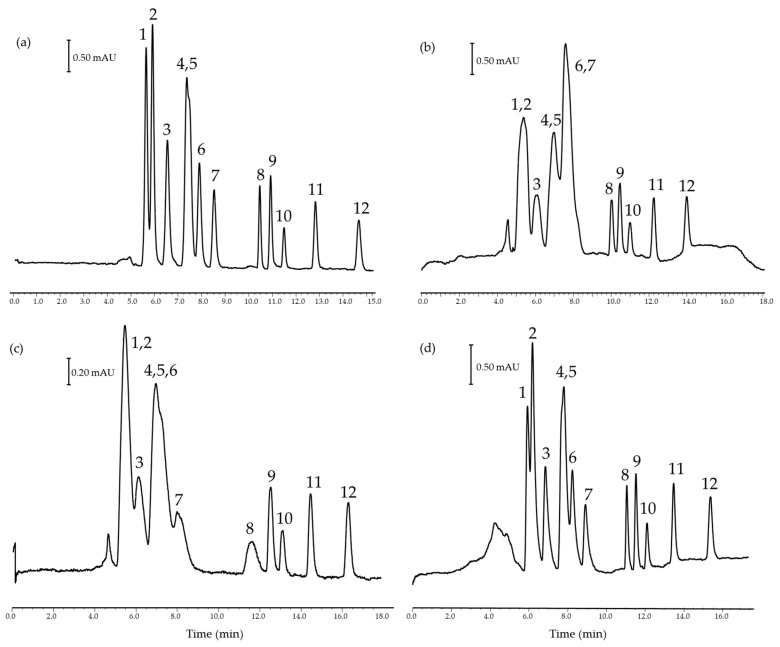
Comparison of the nano-LC-UV separation of a mixture of standard analytes in (**a**) mobile phase ACN/water, 50/50 (*v*/*v*), (**b**) methanol and (**c**) ACN, and (**d**) ACN with a previous water plug of 10 s. Peak identification: (1) Phenol, (2) BPA, (3) 2,4-DMP, (4) 2,3,6-TMP, (5) 4-TBP, (6) 4-SBP, (7) 4-TAP, (8) 4-HexP, (9) 4-TOP, (10) 4-HepP, (11) 4-OP, (12) 4-NP.

Considering these challenges, a combined capillary column was evaluated against the pure C_18_ stationary phase. The concept behind this mixed phase is to incorporate a sort of pre-column with a more apolar C_30_ phase, which enables the compatibility of HNAES with a reverse phase by injecting them in an ACN dilution phase. Several modifications were evaluated in both stationary phases, including mobile phase composition, injection solvents, type, and time of injection, but despite all efforts, the stationary phase containing C_30_ presents some challenges. Firstly, the preparation of this column is more complex than the one of single packaging. Then, the characteristics of the column hinder the chromatographic performance in terms of efficiency, peak shape, resolution, and analysis time. Therefore, XBridge C_18_ was selected for further studies. Some examples of varying injection solvents can be shown in Figure 2.

If analytical standard prepared in mobile phase ACN/water, 50/50, *v*/*v* (Figure 2a) and others in 100% methanol (Figure 2b) or ACN (Figure 2c) are compared, it can be seen how the more polar analytes (phenol > BPA > 2,4-DMP > 2,3,6-TMP, > 4-TBP > 4-SBP > 4-TAP) are strongly influenced by the injection conditions losing the chromatographic resolution and peak efficiency. In contrast, the less polar compounds are little influenced (4-TOP > 4-HepP > 4-OP > 4-NP). In the intermediate region, 4-HexP is influenced as the elution strength of the solvent is modified. In this regard, changes in the chromatographic profile of this compound are more affected by ACN standards than by methanol ones. In addition, when a water plug prior to the sample injection of a pure ACN standard is performed (Figure 2d), it considerably improves the efficiency and resolution of all analytes, being much more like the standard in the mobile phase (Figure 2a) than in any of the other conditions (Figure 2b,c). Therefore, based on the improvement of the analytical signal and chromatographic performance, the last condition was selected.

**Figure 3:**
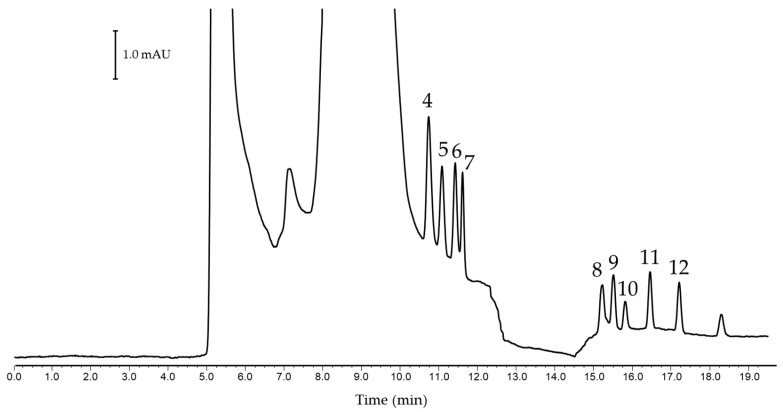
Nano-LC-UV separation of a standard containing 10% (*v*/*v*) of (-)-menthol:*trans*-cinnamic acid HNAES in ACN. Peak identification: (4) 2,3,6-TMP, (5) 4-TBP, (6) 4-SBP, (7) 4-TAP, (8) 4-HexP, (9) 4-TOP, (10) 4-HepP, (11) 4-OP, (12) 4-NP.

To simulate the behavior of an HNAES extract, analytical standards containing 10% (*v*/*v*) of HNAES in ACN were prepared (see Figure 3). As can be seen, the chromatographic profile drastically changes in two different ways. In the first place, the initial three compounds remain within this characteristic band of some NADES, which in some cases can be observed in the tail of the band. However, HNAES manages not only to improve the peak efficiency of the less polar compounds but also the resolution without altering the retention times. Lastly, the latter analytes are again unaffected.

### 3.2. HNAES Temperature-Controlled LLME-Nano-LC-UV Method for the Analysis of Tea Infusion Samples

**Figure 4:**
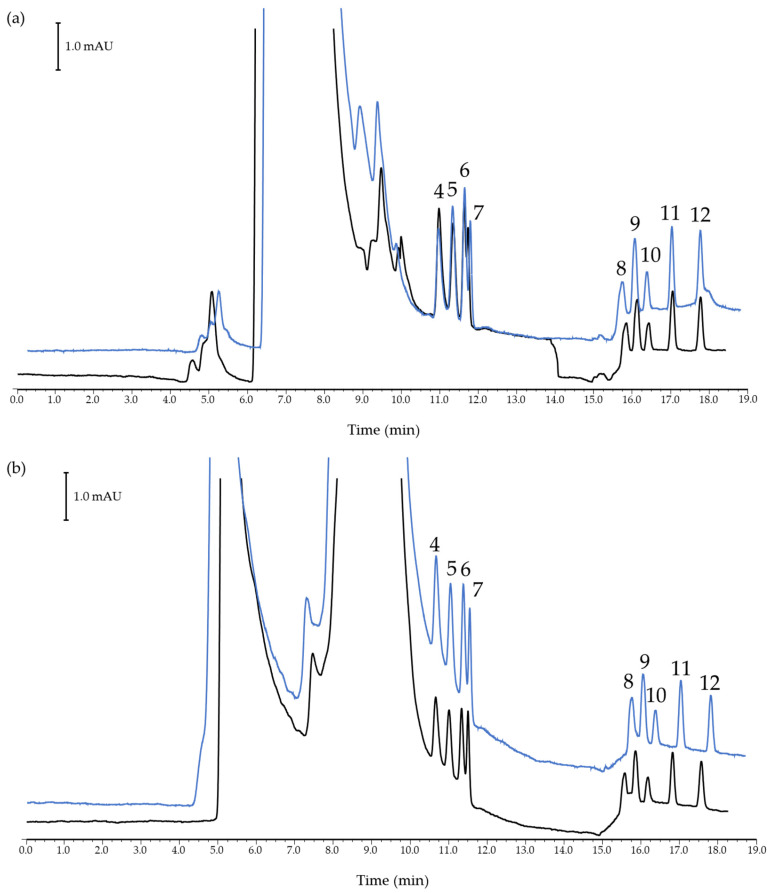
Nano-LC-UV chromatograms of a tea sample extract spiked with target analytes before (blue) and after (black) the microextraction step using HNAES of (-)-menthol combined with (**a**) vanillin and (**b**) *trans*-cinnamic acid. Peak identification: (4) 2,3,6-TMP, (5) 4-TBP, (6) 4-SBP, (7) 4-TAP, (8) 4-HexP, (9) 4-TOP, (10) 4-HepP, (11) 4-OP, (12) 4-NP.

Once it was seen that HNAES favored chromatographic development in some way while finding limitations on the use of UV detection, extraction was performed in tea-based drinks. In this regard, the two HNAES prepared in this work were evaluated. To assess the extraction capacity of these solvents, a recovery study was performed comparing the peak areas of analytes in the samples spiked at the beginning and end of the extraction process. The graphical results are shown in Figure 4. As can be seen, comparing the extraction (in blue) with the matrix-spiked (in black), the ability to extract these HNAES is promising.

However, as in similar cases [13,51], broadband appeared in the first half of the chromatogram, hindering the determination of three of the target analytes. In addition, one of the limitations of the analysis of contaminants such as alkylphenols and BPA is that their sensitivity decreases as the wavelength increases. Thus, this approach could be further optimized for laboratories possessing more sensitive and selective analysis systems, such as mass spectrometry detectors. In these instances, tandem mass spectrometry systems, in particular, could significantly aid in resolving this type of problem by providing detailed information on mass-to-charge ratios and compound fragmentation patterns. Nevertheless, for the case of compounds that are separated by reverse phase systems but that are not friendly to water and can be easily determined by conventional systems such as UV and fluorescence detectors, this approach is very promising.

Afterward, in order to evaluate the applicability of the LLME-nano-LC-UV method, the limits of the method were calculated with both HNAES. Limits of detection (LODs) of the method defined as the concentration which provided a signal-to-noise ratio of 3, ranged between 0.034 and 0.107 μg/mL for (-)-menthol/vanillin and 0.045–0.125 μg/mL for (-)-menthol/*trans*-cinnamic acid HNAES. The limits of quantification (LOQ) of the method, defined as the concentration that provided a signal-to-noise ratio of 10, were experimentally checked by the analysis of samples spiked at this concentration level. In addition, in order to study the extraction efficiency of these solvents, a recovery study was performed for both HNAES at the LOQ level by comparing the peak area of three matrices spiked at the beginning of the procedure with one spiked at the end. The results obtained, as can be seen in Figure 4, showed good extraction capacity with recovery values in the range of 86–115% with relative standard deviations below 20% in all cases. In the case of more polar analytes, including phenol, BPA, and 2,4-DMP, the broadband severally affects their measurement since these compounds appear in the tail of the DES band or are covered by it. Thus, as previously indicated, it can be solved, for instance, by using a more selective/specific detection system.

### 3.3. Greenness and Blueness Assessment

**Figure 5:**
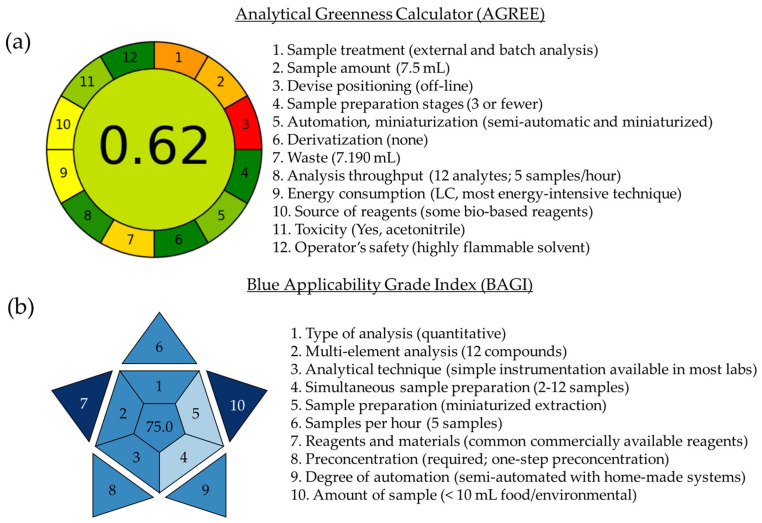
Results obtained by the (**a**) AGREE and (**b**) BAGI metric tools of the developed HNAES-temperature-controlled LLME-nano-LC-UV method.

In this work, to easily compare the different metric tools and evaluate the results obtained, a comparison specifically focusing on the AGREE [61] and BAGI [63] metric tools was conducted. The results are shown in Figure 5.

The AGREE method [61] assesses the environmental friendliness of an analytical methodology through the 12 principles of green analytical chemistry. The outcome is depicted in a pictogram divided into 12 segments, each representing a principle, with a color gradation from dark green to red (indicating adherence to green principles to non-compliance with them). At the center of the pictogram, the evaluation is reflected on a scale ranging from 0 to 1, which indicates an ideal analytical procedure. As can be seen in Figure 5a, the developed methodology based on a temperature-controlled LLME with HNAES as extraction solvents and nano-LC-UV system as determination technique is characterized by external sample preparation and batch analysis with a reduced number of steps (principle 1) and a relatively low volume of sample (7.5 mL, principle 2). As a negative aspect, the analytical is performed in off-line mode, which goes against principle 3, but the number of sample preparation steps is very reduced in favor of principle 4. The developed method has been considered semi-automatic with miniaturized sample preparation using an extraction volume of 100 μL (principle 5) and in the absence of derivatization prior to analysis (principle 6). Although it does not represent a particularly large waste, considering that the sample, extractant, and dilution solvent, among others, are included, principle 7 establishes that a volume of 7.190 mL implies a large volume of analytical waste. In addition, our university has a plan for residue management. The number of compounds analyzed in a single run is twelve, and it is possible to analyze five samples per hour (principle 8). The most energy-intensive system is the nano-LC system (principle 9). In addition, the reagents used for the preparation of HNAESs and extraction components are bio-based (principle 10). As in the case of principle 7, the use of a very low volume of toxic solvents goes against principle 11. In this case, it was considered a volume of 190 µL considering the use of (-)-menthol, vanillin, and *trans*-cinnamic acid, which present the exclamation pictogram, and ACN as dilution solvent prior to analysis. Keeping this in mind, with the proposed method, a punctuation of 0.62 out of 1.00 was achieved, which means good compliance with the green analytical chemistry principles. Despite the good results obtained, some aspects of the AGREE tool represent a very difficult task. The objective of maximizing the number of determinations across diverse compound families in samples of varying nature is only achievable by employing systems that consume high energy and require extensive sample pretreatment, making direct analysis a challenging approach.

Finally, the BAGI [63] serves as a metric tool to measure the practicality and suitability of the method. Complementing green metrics, this tool incorporates white analytical chemistry principles. Its evaluation results are presented visually using a blue asteroid pictogram divided into ten segments, with the final score (25–100) displayed centrally. This approach enables the assessment of the method’s effectiveness and practicality. As depicted in Figure 5b, the method’s strengths and weaknesses are illustrated with a final score of 75.0 out of 100, indicating that the method is indeed practical, surpassing the recommended threshold of 60 points. This evaluation remains consistent for both HNAES, owing to their widespread commercial availability. Notably, the method could be further enhanced. Firstly, adopting a mass spectrometry system for result confirmation (principle 1) is recommended. Secondly, increasing the capacity for simultaneous sample preparation (principle 4) and downsizing the extraction system (principle 5) would be advantageous. Despite these areas for improvement, the developed methodology stands as a straightforward and cost-effective analytical approach.

## 4. Conclusions

A new methodology based on an HNAES temperature-controlled LLME as an extraction method and a nano-LC-UV detection as a determination system has been developed for the analysis of twelve xenobiotic compounds in tea beverages. The investigation focused on optimizing the separation process by considering various factors such as stationary phase, mobile phase, and injection solvent compositions, with the best results achieved utilizing an XBridge C_18_ capillary column under on-column focusing step gradient mode. For the injection solvent, acetonitrile was selected as the most suitable for HNAES dilution and analytes solubilization.

The HNAESs prepared based on (-)-menthol with vanillin or *trans*-cinnamic acid were reported and characterized for the first time. DSC experiments demonstrated that these solvents are ideal liquid mixtures with slight negative deviation to ideality. As extraction solvents in aqueous samples such as tea infusion samples, these HNAESs resulted in promising extractants. In addition, their presence in the injection solvent improves the chromatographic performance despite the high elution strength conferred by using ACN as a single-dilution solvent. This aspect may result in the exploitation of another field of application in which this type of solvent can be used to improve chromatographic development.

Finally, the greenness and blueness of the developed methodology were evaluated using the AGREE and BAGI metric tools, obtaining scores of 0.62 out of 1.00 and 75.0 out of 100.0, respectively. These good results suggest that despite the fact that there are many aspects that can be improved in the method, in general terms, it can be considered a good fulfillment of the green principles while ensuring its functionality.

## Figures and Tables

**Table 1:** Step gradient applied for the separation of alkylphenols and BPA in tea drinks.

Stationary Phase	Column Conditioning	Step Gradient (Time; Composition)
XBridge^®^ C_18_	50/50, ACN/H_2_O, *v*/*v*	4.0 min; 70/30, ACN/H_2_O, *v*/*v*
		12.0 min; 100% ACN

Injection volume: 40 nL; flow rate: 240 nL/min.

**Table 2:** Components used for the preparation of HNAES and their melting properties.

Compound	Structure	T_m_ (K)	Δ_m_h (KJ/mol)	Reference
(-)-Menthol	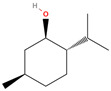	315.7	12.9	[54]
Vanillin	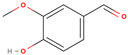	355.4	22.4	[55]
*trans*-Cinnamic acid	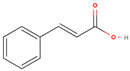	404.8	22.6	[56]

T_m_: melting point; Δ_m_h: enthalpy of fusion.

## Data Availability

The original contributions presented in the study are included in the article/Supplementary Material, further inquiries can be directed to the corresponding authors.

## Supplementary Materials

The following supporting information can be downloaded at: https://www.mdpi.com/article/10.3390/foods14010028/s1, Figure S1: Solid–liquid phase diagrams predicted by the ideal liquid phase model (blue dashed lines) and experimental solid–liquid equilibrium data measured using DSC (orange full lines) of mixtures formed by (-)-menthol and (a) vanillin and (b) *trans*-cinnamic acid. The green horizontal dashed line represents the working temperature (323.15 K); Figure S2: FT-IR spectra of (a) (-)-menthol, (b) vanillin, and (c) (-)-menthol/vanillin HNAES; Figure S3: FT-IR spectra of (a) (-)-menthol, (b) *trans*-cinnamic acid, and (c) (-)-menthol/*trans*-cinnamic acid HNAES.
