# Supplementary material for: Natural Eutectic Solvent-Based Temperature-Controlled Liquid–Liquid Microextraction and Nano-Liquid Chromatography for the Analysis of Herbal Aqueous Samples
Source: Foods. 2024 Dec 25;14(1):28. doi: 10.3390/foods14010028 (PMC11720319; doi:10.3390/foods14010028)
Supplement: Supplementary file 1 [file foods-14-00028-s001.zip › foods-3363696-supplementary.pdf]

foods

MDPI

Supplementary Material

Natural Eutectic Solvent-based temperature-controlled liquid-liquid microextraction and nano-liquid chromatography for the analysis of herbal aqueous samples

Álvaro Santana-Mayor [1,2,*], Giovanni D'Orazio [2], Miguel Ángel Rodríguez-Delgado [1] and Bárbara Socas-Rodríguez [1,*]

[1] Departamento de Química, Área de Química Analítica, Facultad de Ciencias, Universidad de La Laguna (ULL), Avenida Astrofísico Francisco Sánchez s/n, 38206 San Cristóbal de La Laguna, Tenerife, Spain; asan-tanm@ull.edu.es (Á.S.-M.); bsocasro@ull.edu.es (B.S.-R.); mrguez@ull.edu.es (M.Á.R.-D.)

[2] Istituto per i Sistemi Biologici (ISB), CNR- Consiglio Nazionale delle Ricerche, Montelibretti, Rome, Italy; giovanni.dorazio@cnr.it (G.D.)

* Correspondence: bsocasro@ull.edu.es (B.S.-R.); Tel.: +34-922-318-036; asantanm@ull.edu.es (Á.S.-M.)

1. Characterization of HNAES

The two new type V HNAES based on vanillin or *trans*-cinnamic acid with (-)-menthol can be characterized through their solid-liquid curves based on the equation (1) [1]:

$$\mathrm{Ln}\ (\chi i \cdot \gamma i) = \Delta_m h_i / R \cdot (1/T_{m,i} - 1/T) \tag{1}$$

where χi is the mole fraction of the component i, γi is the activity coefficient, $\Delta_m h_i$ and $T_{m,i}$ are the enthalpy and melting temperature of the pure compound i, R is the universal gas constant, and T is the absolute temperature of the system. In this case, since the temperature is not far from the melting point of the pure compounds, the molar heat capacity associated with the classical thermodynamics equations has been neglected. In addition, based on the ideal liquid phase model, the system is considered thermodynamically ideal and thus, the activity coefficients are considered as unit. Therefore, the equation above is expressed as the equation (2):

$$\mathrm{Ln}\ (\chi i) = \Delta_m h_i / R \cdot (1/T_{m,i} - 1/T) \tag{2}$$

Following this criterion, solid-liquid curves of both HNAES were obtained as the combination of the ideal solubility curves of the pure components (data shown in Table 2). The results of the predicted phase diagrams are shown in Figure S1.

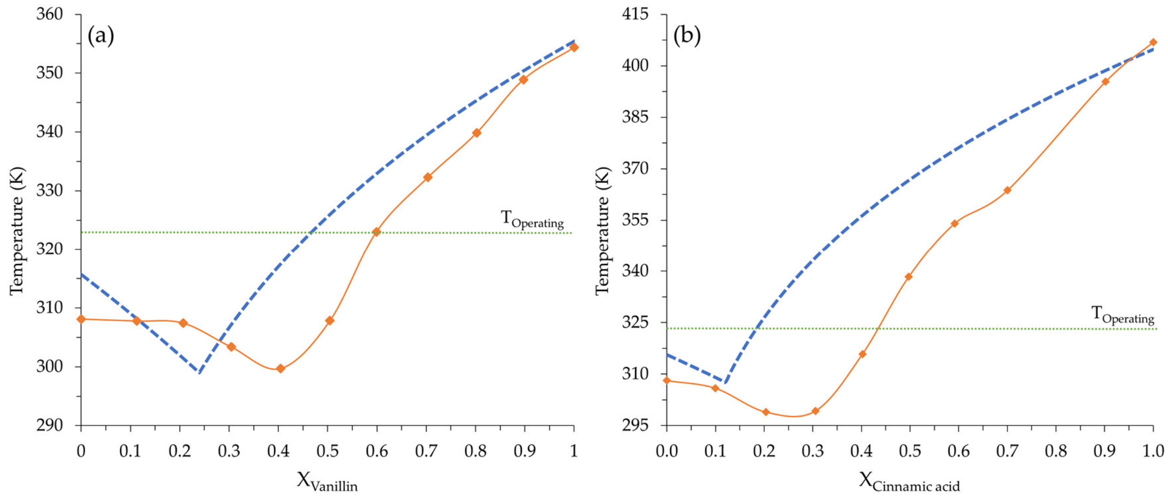

Figure S1. Solid-liquid phase diagrams predicted by the ideal liquid phase model (blue dashed lines) and experimental solid-liquid equilibrium data measured using DSC (orange full lines) of mixtures formed by (-)-menthol and (a) vanillin and (b) *trans*-cinnamic acid. The green horizontal dashed line represents the working temperature (323.15 K).

As can be seen, the predicted eutectic temperatures are below the operating temperature. In addition, experimental data showed an almost ideal liquid behavior with small negative deviations to ideality for both mixtures with a depression in the freezing temperature close to 293.15 K (room temperature) in a wide range of compositions. Based on that, for (-)-menthol:vanillin, a mole fraction of 0.76:0.24 was set (3:1, n/n) while 0.88:0.12 (7:1, n/n) was set for the one of (-)-menthol:*trans*-cinnamic acid HNAES.

Afterward, FT-IR analysis of (-)-menthol, vanillin, and *trans*-cinnamic acid, as well as the ones of the newly synthesized HNAES, was performed to investigate the formation of the prepared solvents. The resulting spectra are shown in Figure S2. The spectrum of (-)-menthol in Fig S2(a) shows the main bands at 3246, 2953-2869, 1460-1446, 1044, and 1025 cm^{-1} that can be associated with the O-H stretching vibration, the C-H stretching, the methyl group bending, and the C-O bond stretching, respectively. Regarding the spectrum of vanillin in Figure S2(b), the bands at 3221, 1661, 1021, and 588 cm^{-1} can be assigned to the stretching vibrations of O-H, C=O, and C-O-C groups, and the torsion of the aldehyde group, respectively. In addition, the FT-IR spectrum of the HNAES formed by both compounds showed a shift of the bands associated with the hydroxyl and carbonyl groups to higher wavenumbers (3340 cm^{-1} and 1672 cm^{-1}, respectively). This shifting can be attributed to the formation of hydrogen bonds between (-)-menthol and vanillin and, therefore, the formation of the HNAES (see Figure S2(c)). These results are in good accordance with previously reported data [2–4].

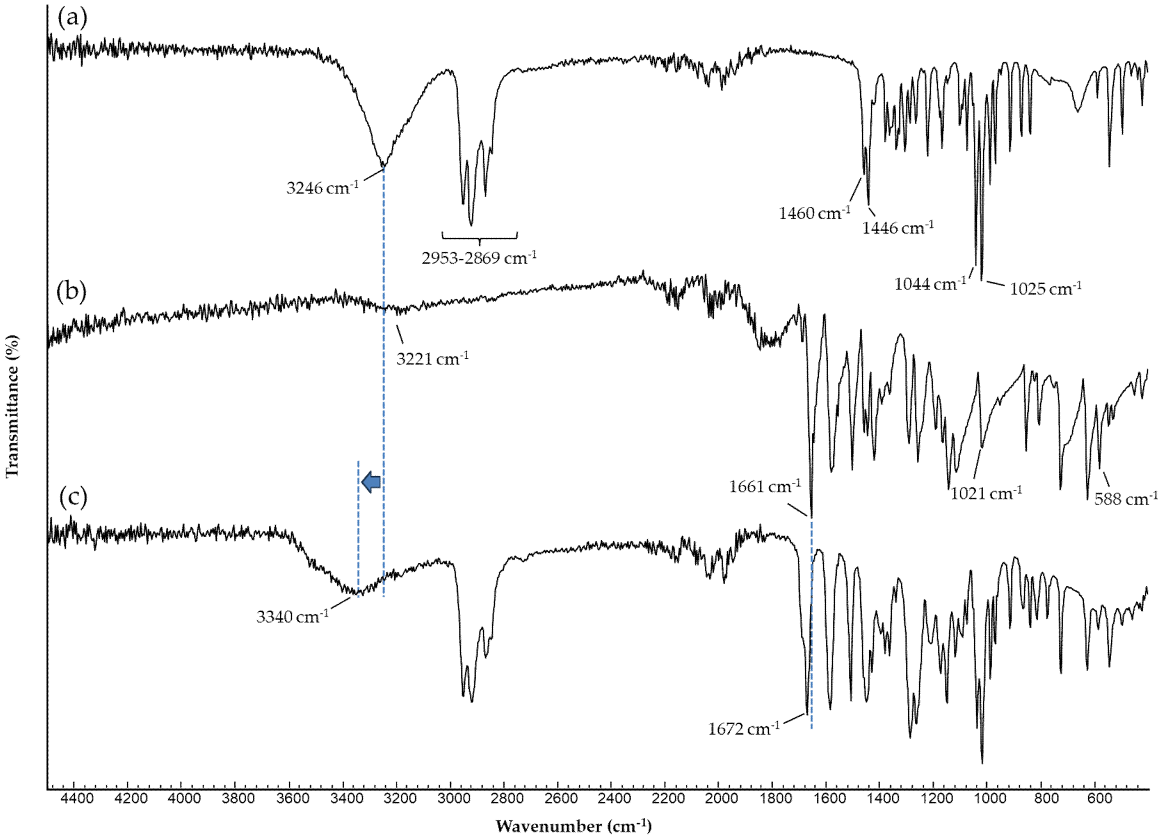

Figure S2. FT-IR spectra of (a) (-)-menthol, (b) vanillin, and (c) (-)-menthol:vanillin HNAES.

FT-IR spectrum of *trans*-cinnamic acid is shown in Figure S3(b). The characteristic band associated with the carboxylic acid appeared at 3060-2560 cm^{-1} as a broad peak. In addition, bands attributed to the C=O, C=C, and C-O stretching vibrations and the O-H bending are presented at 1680, 1630, 1281, and 976 cm^{-1}, respectively [5,6]. As in the case of the HNAES of (-)-menthol with vanillin, Figure S3(c) of the *trans*-cinnamic acid HNAES shows a shift of the hydroxyl band to a higher wavenumber (3360 cm^{-1}). Also, carbonyl and C=C vibration bands moved to higher frequencies (1969 cm^{-1} and 1637 cm^{-1}, respectively). These shifts can be associated with the formation of hydrogen bonds between both compounds, confirming the formation of the solvent.

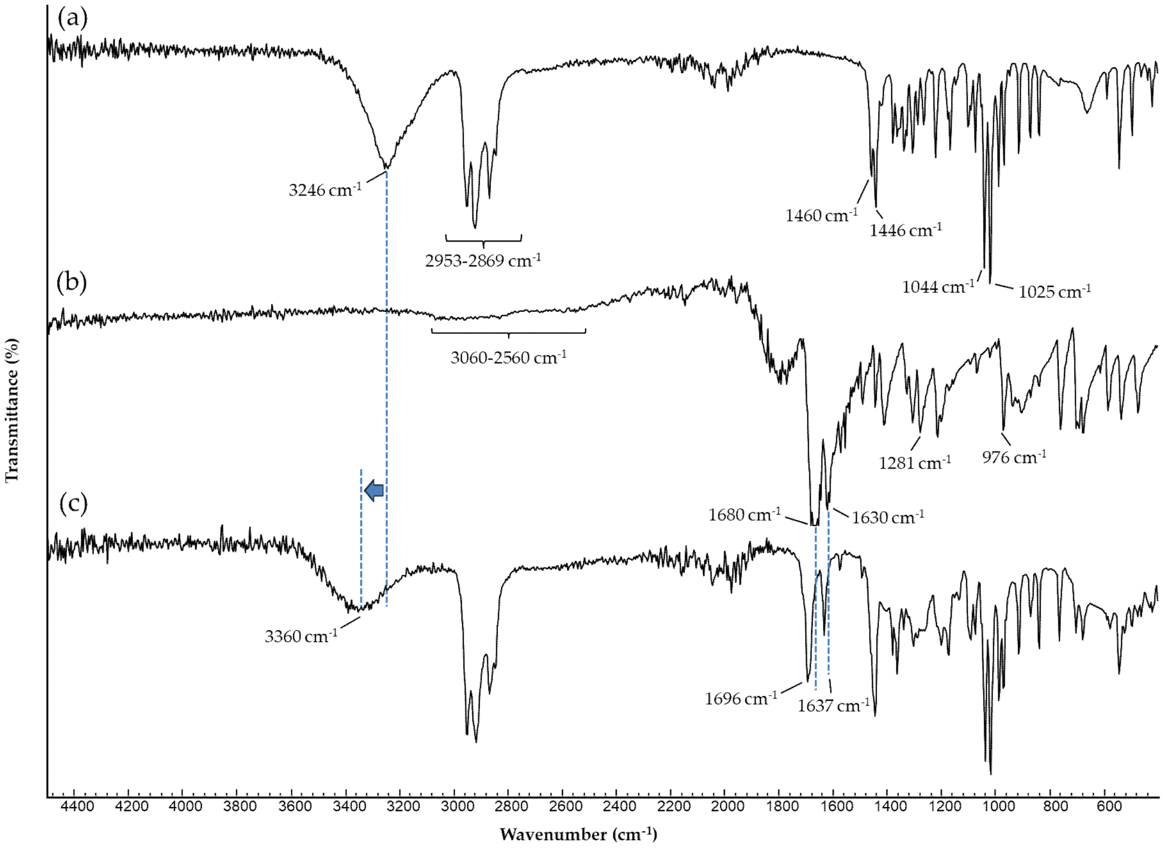

54

Figure S3. FT-IR spectra of (a) (-)-menthol, (b) *trans*-cinnamic acid, and (c) (-)-menthol:*trans*-cinnamic acid HNAES. 55

56

References

1. Abranches, D.O.; Martins, M.A.R.; Silva, L.P.; Schaeffer, N.; Pinho, S.P.; Coutinho, J.A.P. Phenolic Hydrogen Bond Donors in the Formation of Non-Ionic Deep Eutectic Solvents: The Quest for Type v Des. *Chem. Commun.* **2019**, *55*, 10253–10256, doi:10.1039/c9cc04846d.
2. Soltani, S.; Sereshti, H.; Nouri, N. Deep Eutectic Solvent-Based Clean-up/Vortex-Assisted Emulsification Liquid-Liquid Microextraction: Application for Multi-Residue Analysis of 16 Pesticides in Olive Oils. *Talanta* **2021**, *225*, 121983, doi:10.1016/j.talanta.2020.121983.
3. Cordeiro, T.; Castiñeira, C.; Mendes, D.; Danède, F.; João, S.; Fonseca, I.M.; Gomes da Silva, M.; Paiva, A.; Barreiros, S.; Cardoso, M.M.; et al. Stabilizing Unstable Amorphous Menthol through Inclusion in Mesoporous Silica Hosts. *Mol. Pharm.* **2017**, *14*, 3164–3177, doi:10.1021/acs.molpharmaceut.7b00386.
4. Mat Hussin, S.A.; Varanusupakul, P.; Shahabuddin, S.; Yih Hui, B.; Mohamad, S. Synthesis and Characterization of Green Menthol-Based Low Transition Temperature Mixture with Tunable Thermophysical Properties as Hydrophobic Low Viscosity Solvent. *J. Mol. Liq.* **2020**, *308*, 113015, doi:10.1016/j.molliq.2020.113015.
5. Batista, R.S. de A.; Melo, T.B.L.; dos Santos, J.A.B.; de Andrade, F.H.D.; Macedo, R.O.; de Souza, F.S. Evaluation of Crystallization Technique Relating to the Physicochemical Properties of Cinnamic Acid. *J. Therm. Anal. Calorim.* **2019**, *138*, 3727–3735, doi:10.1007/s10973-019-08455-7.
6. Shan, Y.; Han, Y.; Fan, C.; Liu, Y.; Cao, X. New Natural Deep Eutectic Solvents Based on Aromatic Organic Acids. *Green Chem. Lett. Rev.* **2021**, *14*, 713–719, doi:10.1080/17518253.2021.2009579.

Disclaimer/Publisher's Note: The statements, opinions and data contained in all publications are solely those of the individual author(s) and contributor(s) and not of MDPI and/or the editor(s). MDPI and/or the editor(s) disclaim responsibility for any injury to people or property resulting from any ideas, methods, instructions or products referred to in the content.
